# Tissue spray ionization mass spectrometry for rapid recognition of human lung squamous cell carcinoma

**DOI:** 10.1038/srep10077

**Published:** 2015-05-11

**Authors:** Yiping Wei, Liru Chen, Wei Zhou, Konstantin Chingin, Yongzhong Ouyang, Tenggao Zhu, Hua Wen, Jianhua Ding, Jianjun Xu, Huanwen Chen

**Affiliations:** 1 Department of Cardiothoracic Surgery to Second Affiliated Hospital of Nanchang University, Nanchang, Jiangxi Province 330006, P. R. China; 2 East China Institute of Technology, Jiangxi Key Laboratory for Mass Spectrometry and Instrumentation, Nanchang, Jiangxi Province 330013, P. R. China

## Abstract

Tissue spray ionization mass spectrometry (TSI-MS) directly on small tissue samples has been shown to provide highly specific molecular information. In this study, we apply this method to the analysis of 38 pairs of human lung squamous cell carcinoma tissue (cancer) and adjacent normal lung tissue (normal). The main components of pulmonary surfactants, dipalmitoyl phosphatidylcholine (DPPC, *m/z* 757.47), phosphatidylcholine (POPC, *m/z* 782.52), oleoyl phosphatidylcholine (DOPC, *m/z* 808.49), and arachidonic acid stearoyl phosphatidylcholine (SAPC, *m/z* 832.43), were identified using high-resolution tandem mass spectrometry. Monte Carlo sampling partial least squares linear discriminant analysis (PLS-LDA) was used to distinguish full-mass-range mass spectra of cancer samples from the mass spectra of normal tissues. With 5 principal components and 30 – 40 Monte Carlo samplings, the accuracy of cancer identification in matched tissue samples reached 94.42%. Classification of a tissue sample required less than 1 min, which is much faster than the analysis of frozen sections. The rapid, in situ diagnosis with minimal sample consumption provided by TSI-MS is advantageous for surgeons. TSI-MS allows them to make more informed decisions during surgery.

In situ identification of tumor margins is crucially important in surgical oncology to obtain confident curative resection and accurate prognosis, as well as to minimize losses to healthy tissues. Lung cancer is one of the most common malignancies worldwide. With the highest rate of morbidity and mortality^1^, squamous carcinoma is the most common lung cancer^2^, and surgery is one of the most effective ways to cure this disease^3^. Identification of tumor margins is particularly important in thoracic surgery to select either lobectomy or pneumonectomy. Currently, tumor margins are established by means of preoperative medical imaging. Tumors are excised within a predefined safety zone or ‘resection margin’, which is defined by the size, location and stability of the tumor^3^,^4^. In some cases, particularly when the tumor is approaching major blood vessels or nerves, the safety zone margin is larger than the normal safety zone margin. Frozen-section histology is the gold-standard method to accurately determine tumor margin during surgery^5^, but this approach suffers from shortcomings^6^. The most critical shortcoming is the complicated workflow of the analysis, including sampling, transferring the sample to a distant histopathology lab, labor-intensive sample processing, investigator dependent analysis of the resulting sections, and verbal communication of the results. Frozen-section histology has a mean turnaround time of 30–40 min, and, quite often, additional samples can be required^5^. Furthermore, during this time the patient remains under anesthesia^7^. Therefore, there is a need for real-time, in situ identification of arbitrary tissue features during surgical, therapeutic, and some diagnostic interventions^8^.

Chemical analysis by mass spectrometry (MS) may be an alternative to standard frozen-section histology, as mass spectrometry can provide rich molecular information on the chemicals on and in the tissue, which can be correlated with pathology^9^,^10^,^11^. Recently, MS analysis of tissue has advanced rapidly and a number of ionization methods have been used to characterize tissue sections without performing chemical extraction prior to analysis. These methods include nanostructure initiator mass spectrometry (NIMS)^12^, secondary-ion mass spectrometry (SIMS)^13^, matrix-assisted laser desorption (MALDI)^14^,^15^, laser ablation electrospray ionization (LAESI)^16^, touch spray mass spectrometry^17^, and desorption electrospray ionization (DESI)^18^, probe electrospray ionization (PESI)^19^,^20^ and internal extractive electrospray ionization mass spectrometry (iEESI-MS)^8^,^11^. These methods compare the distribution of particular compounds (e.g., phospholipids, proteins) with known patterns and characteristics of diseased versus healthy tissue. Some other ambient ionization methods, such as electrospray laser desorption ionization (ELDI)^21^ and extractive electrospray ionization (EESI)^11^ can also be used for direct tissue analysis without sample preparation^22^.

Several MS methods have been demonstrated suitable for the direct and simple analysis of tiny tissue samples^23^, such as those obtained by needle biopsy. In some experiments, a tissue section was placed on paper^24^ or on a wooden tip^25^, which were wetted with solvent. A large electrical potential was applied to create electrospray that contained small molecules extracted from the tissue sample. More recently, Graham Cooks and coworkers^10^ performed MS analysis of animal tissue samples from biopsy needle. Electrospray ionization directly from the sample on the tip of biopsy needle was shown to provide abundant and highly specific molecular information via MS analysis. Lipid profiles have been suggested to be particularly useful for tissue characterization.

In the present work, we extend the application of tissue spray ionization (TSI) MS to identify lung squamous cell carcinoma in humans. Tiny tissue pieces (1 mm^3^) were analyzed from the tip of an acupuncture needle. A high voltage was applied to the needle, and to the surface of the tissue. Spectra of high-peak abundances were detected, and characteristic ions were directly observed. Using Monte Carlo sampling and partial least squares linear discriminant analysis (PLS-LDA) of TSI mass spectra, the accuracy of cancer identification in matched tissue samples reached 94.42%. The described experimental procedure is comparable with paraffin-section pathology results.

## Results

### TSI-MS of lung tissue

**Figure 1:**
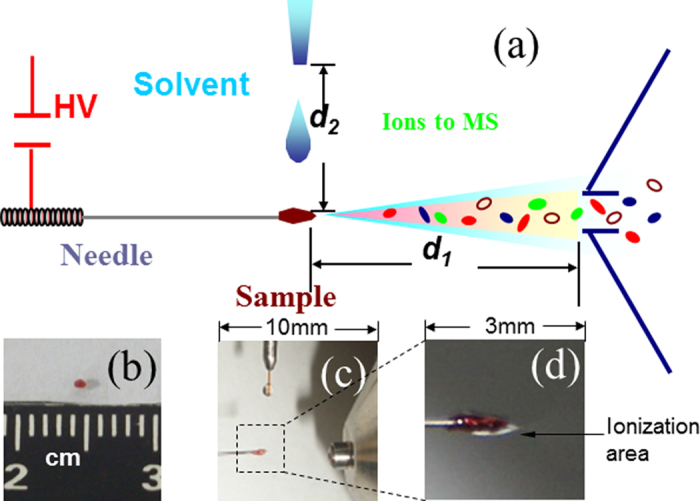
(**a**) Schematic showing direct tissue spray ionization analysis from a tissue loaded onto the sharp tip of an acupuncture needle. High voltage is applied (HV: +3.5 kV) to the other end of the needle. *d*_*1*_: 5 mm; *d*_*2*_: 5 mm. (**b**) Tissue size is about 1 mm^3^. (**c**) Tissue held by the needle without added spray solvent. (**d**) A Taylor cone forms on the tissue after applying both high voltage and spray solvent.

**Figure 2:**
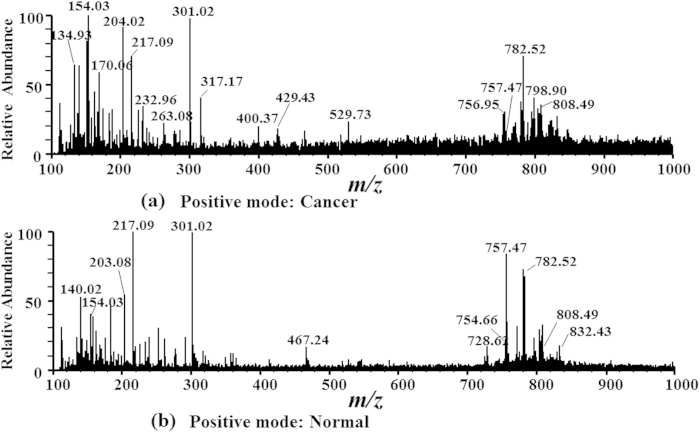
Spectra obtained from human lung cancer and normal lung tissue. (**a**) Cancerous tissue in positive ion mode with main peaks at *m/z* 154.03, *m/z* 170.06, *m/z* 798.90, *m/z* 808.49, and *m/z* 832.43, and so on. (**b**) Normal tissue in positive ion mode with main peaks at *m/z* 203.08, *m/z* 757.47 and *m/z* 782.52, and so on.

A Taylor cone forms on the tissue (Fig. 1) when both the solvent drop and the high voltage were applied. As shown in Fig. 1, the tissue serves as the spray tip in this ionization experiment, and the solvent extracts molecules^9^ in the tissue and carries them to the tip of the Taylor cone where charged droplets containing analyte molecules are formed. This method works particularly well for very small samples (<1 mm^3^). A small volume of solvent (2 *μL* methanol) is used, and the spray period lasts approximately 10 s (Fig. S-1). This time is sufficient to acquire high-quality mass spectral data containing MS and MS/MS scans. The analysis can be repeated five times with the same sample by using different solvents. The total ion intensity can vary about 30% during each spray period over the course of 3 minutes; however, the relative intensities were found to be highly reproducible (Fig. S-1). This experiment was also repeated using samples from different extractions of the same tissue. The variation in total ion intensity was still within one order of magnitude, with similar relative intensities obtained for each analysis. The relative standard deviation (RSD) was obtained by measuring the abundance of ions at *m/z* 782.5, 808.5 and 832.5 (MS stage) of the tumor tissues, and the RSDs were 18.17%, 18.84% and 17.83%, respectively (Table S-1). Tandem mass spectrum and Collision Induced Dissociation (CID) identified a wide range of fatty acids and phospholipids in the tissues examined (Fig. 2). The concentrations of these chemicals in each sample has no related to any clinical parameter, such as, TNM stage, tumor placement in the lung, gender, age or smoking history.

The cell structure inside the samples was altered after MS analysis. Before MS analysis, cells were closely packed with no significant space between cells (Fig. S-2 b, d), but after MS analysis, some cells were collapsed, and the space between the cells could be clearly observed (Fig. S-2 c, e). The cells lysed.

### Differentiation of cancerous tissue from noncancerous tissue by PLS-LDA

**Figure 3:**
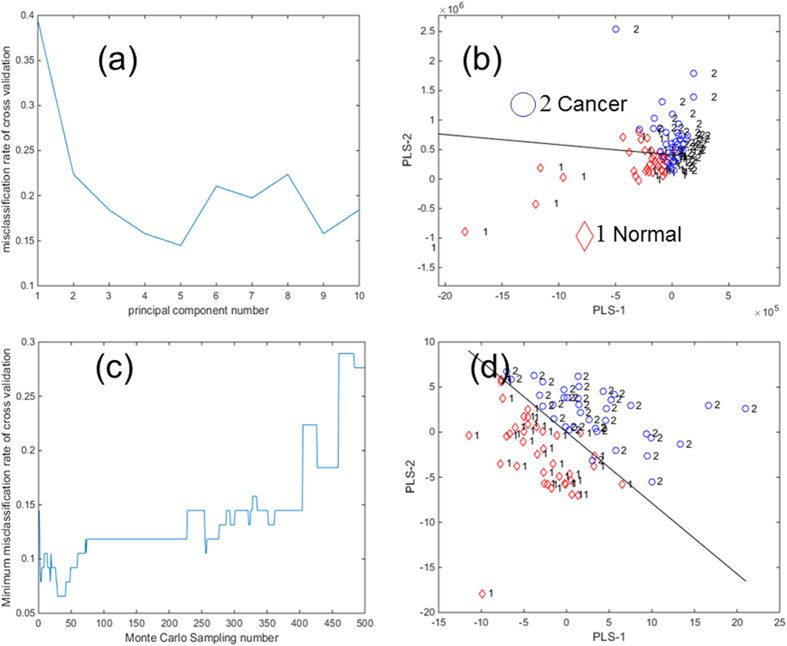
Statistical analysis of mass spectra; Monte Carlo sampling and partial least squares linear discriminant analysis (PLS-LDA) plots of all mass data. When the principal component number is 5 the misclassification rate is (**a**) 14.47%. (**b**) PLS-2 versus PLS-1 plot from –20 to 10. (**c**) With 30-40 Monte Carlo samplings (**c**), the best discrimination accuracy was 94.42% (**d**) PLS-2 versus PLS-1 plot from –15 to 15.

PLS-LDA of the spectra recorded shows a clear distinction between the samples from human lung squamous cell carcinoma tissue (cancer) and adjacent normal lung tissue (normal). A strong sampling signal was observed with good reproducibility as the solvent dripped onto the tiny tissue sample (Fig. 2). At a flow rate of 4 μL/min, the solvent dripped a drop about every 30s. Analyte signals were particularly abundant in the *m/z* 700–900 range in positive ion mode. A total of 380 data points from 190 pairs of matched samples (five points per sample) were interpreted in the score graphs of the PLS-LDA. After PLS dimension reduction, the first two-dimensional LDA ingredient was used in the scoring matrix discriminant analysis. When the principal component number is 5 (Fig. 3a), the best discrimination accuracy obtained was 85.53% and the misclassification rate was 14.47% (Fig. 3b). Competitive adaptive reweighted sampling (CARS)^25^ was used for variable selection. With different variable selection, the misclassification rate of cross validation varied from 0.1447 to almost 0.0658. When a Monte Carlo sampling was used 30 – 40 times (Fig. 3c), the best discrimination accuracy obtained was 94.42%, and the misclassification rate was 6.58% (Fig. 3d).

### Preliminary study of lipids specific to lung squamous cell carcinoma

**Figure 4:**
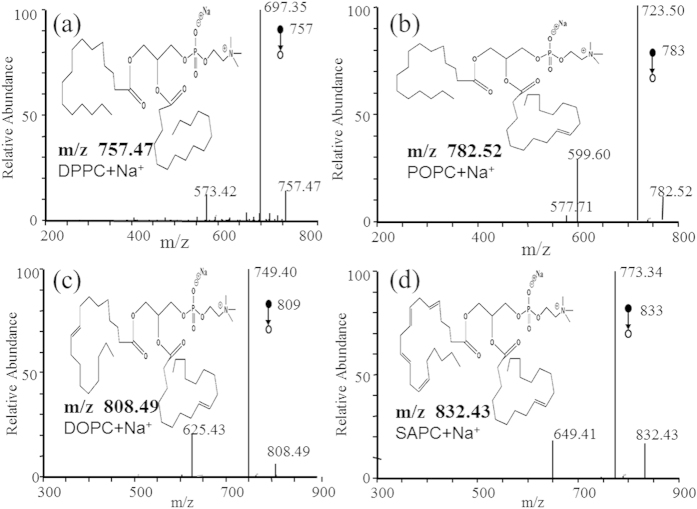
Product ion mass spectra used in multiple reactions monitoring for: (**a**) dipalmitoyl phosphatidylcholine, DPPC + Na^+^ (precursor ion *m/z* 757.47), (**b**) 1-palmityl 2-oleoyl phosphatidylcholine, POPC + Na^+^ (precursor ion *m/z* 782.52), (**c**) 2-oleoyl phosphatidylcholine, DOPC + Na^+^ (precursor ion *m/z* 808.49), and (d) stearoyl phosphatidylcholine, SAPC + Na^+^ (precursor ion *m/z* 832.43).

In the positive ion mode, the dominant species in the *m/z* range 700–900 are phospholipids, and the main peaks are identified as protonated and sodium adducts of phosphatidylcholine (PC). A systematic difference was clearly observed for TSI-MS fingerprints collected from cancerous and normal tissues. The relative abundances of *m/z* 154.03 (5.75 ± 2.39 *vs* 3.26 ± 1.53, *P < *0.05), *m/z* 170.06 (3.65 ± 2.37 *vs* 1.21 ± 0.56, *P < *0.05), *m/z* 798.90 (4.69 ± 2.31 *vs* 2.35 ± 0.87, *P < *0.05), *m/z* 808.49 (4.95 ± 1.56 *vs* 3.82 ± 1.36, *P < *0.05), and *m/z* 832.43 (3.96 ± 1.53*vs* 2.61 ± 1.15, *P < *0.05) were greater in cancerous tissue, and the relative abundances of *m/z* 203.08 (1.59 ± 0.84 *vs* 3.56 ± 2.43, *P < *0.05), *m/z* 757.47 (2.59 ± 1.08 *vs* 6.97 ± 2.34, *P < *0.05) and *m/z* 782.52 (3.39 ± 1.54 *vs* 4.67 ± 1.26, *P < *0.05) were higher in normal tissue (Fig. 2). Identified complex lipids include dipalmitoyl PC (DPPC + Na^+^ *m/z* 757.47), 1-palmityl 2-oleoyl PC (POPC + Na^+^ *m/z* 782.52), 2-oleoyl PC (DOPC + Na^+^ *m/z* 808.49), and arachidonic acid-stearoyl PC (SAPC + Na^+^ *m/z* 832.43, Fig. 4).

## Discussion

Our results demonstrate that TSI-MS enables direct chemical characterization of the interior of lung squamous cell carcinoma samples without any pretreatment. The analytical performance of TSI has two advantages over DESI^18^, paper spray^24^, and wooden tip spray^25^ in both sensitivity and the number of detected compounds. Overall, TSI-MS revealed more signals for lung tissue samples (Fig. 2). Because MS analysed the cells, considerable contribution of chemicals extracted from the cytoplasm in addition to those chemicals extracted from the surface can be expected in the observed mass spectra^24^,^25^.

The PLS-LDA indicated that the main chemicals in cancer and normal lung tissue were similar and only a small fraction of compounds was unique for consideration as a biomarker. With PLS dimension reduction, the discrimination accuracy obtained was 85.53% and the misclassification rate was 14.47%. But when Monte Carlo sampling was repeated 30–40 times, the best discrimination accuracy obtained was 94.42% and the misclassification rate was 6.58%. This means that combining TSI-MS and PLS-LDA allows rapid differentiation of cancerous and noncancerous lung tissue with an accuracy rate of >94%.

As demonstrated by the results described above, lipid profiles were readily obtained from tissue in positive ion mode. Pulmonary surfactant is biphasic and is derived from the lamellar inclusions of type II alveolar epithelial cells. The biophysical properties of the lining layer are largely due to a series of phospholipids (78%) and surfactant-specific proteins (2%). In 1987, Jablonka *et al.*^27^ observed the composition of pulmonary surfactant phospholipids and fatty acids of lung cancer patients, and, as it is well known that smoking is the leading correlated condition of lung cancer, he found that smoking lowered PC and phosphatidylglycerol in lung tissue. In this study, we did not find any relation between smoking history and pulmonary surfactant.

Type II alveolar epithelial cells are a type of lung stem cell^28^. Also, the association between genetic variations in surfactant protein D and lung cancer was found in a Japanese patient cohort^29^. Furthermore, Sin *et al*. suggested that pro-surfactant protein B can serve as a biomarker for lung cancer prediction^30^. Eberlin *et al*. also showed that the lipid profile is useful for diagnosing cancer and, in some cases, for recognizing its stage^31^. However, these three studies were mostly focused on gene and protein levels. No evidence was presented for the direct relationship between pulmonary surfactant and lung squamous cell carcinoma in these studies. DPPC + Na^+^ (*m/z* 757.47), POPC + Na^+^ (*m/z* 782.52), DOPC + Na^+^ (*m/z* 808.49), and SAPC + Na^+^ (*m/z* 832.43) are the main components of pulmonary surfactant. In this study, we provide evidence that there are significant concentration differences in pulmonary surfactant between lung squamous cell carcinoma and normal tissues: DPPC and POPC decreased but DOPC and SAPC increased in lung squamous cell carcinoma tissue relative to normal tissue, respectively. More research is required to confirm that type II alveolar epithelial cells transforming into cancer stem cells are the cause of these pulmonary surfactant differences.

In conclusion, our data shows that the direct examination of tissues using TSI-MS analysis can distinguish lung squamous cell carcinoma tissue from normal lung tissues with high reliability. Using tandem MS, we were able to identify various endogenous chemicals in the tested tissues, from small molecules to phospholipids, which can be used for lung squamous cell carcinoma diagnosis. TSI-MS has the necessary characteristics for the surgical theater, such as high speed, simplicity and automation of analysis. MS analysis of tissue biopsies could provide surgeons with critical and previously unavailable information, which could rapidly guide surgical resection and improve treatment of patients with pulmonary cancer^8^.

## Methods

### Sample collection

This study was approved by the Medical Ethnics Committee in the Hospital Institutional Review Board of the Second Affiliated Hospital to Nanchang University, Nanchang, P. R. China. Written informed consent was obtained from all the patients in this study. All clinical investigations were conducted according to the principles expressed in the Declaration of Helsinki. The criteria for patient enrollment in this study were the diagnosis of non-small cell lung cancer confirmed by pathology (Fig. S-2), absence of accompanying malignancies, absence of other lung diseases, and no history of preoperative chemotherapy or radiotherapy. In total, 38 male patients enrolled in this study, with a median age of 60 (40 – 72 years). The group included 3 well–differentiated, 20 moderately–differentiated and 15 poorly–differentiated squamous cell carcinoma cases. In the group, Stage I was observed in 3 cases, I b was observed in 7 cases, II a was observed in 11 cases, II b was observed in 6 cases, IIIa was observed in 10 cases and IIIb was observed in 1 case with the new lung cancer staging system^2^. Every patient sample included two matched pairs, namely, cancer tissue (cancer) and adjacent normal lung tissue (≥5 cm away from the tumor; normal; Fig. S-2). Tissues were collected within 5 min by a trained surgeon (Fig. S-2) and stored at –80 °C. Samples were thawed to 4 °C before TSI-MS analysis.

### Tissue spray ionization mass spectrometry

A small piece of lung tissue (about 1 mm^3^; Fig. 1b), was cut using a disposable sterilized surgical blade (Cardinal Health, Dublin, OH, USA), and directly loaded onto the tip of an acupuncture needle (Fig. 1c). The rest of the sample was verified pathologically (Fig. S-2) using hematoxylin and eosin (H&E) staining.

Experiments were done on a commercial LTQ-XL mass spectrometer (Thermo Fisher, USA) equipped with a homemade ion source for tissue sampling (Fig. 1a). This equipment mainly consists of three parts: tissue spray, extraction solvent, and the LTQ-XL (Fig. 1c). The spray tip was the sharp end of a disposable, sterilized acupuncture needle (0.35 mm × 75 mm, stainless steel; Hanyi TCM, Beijing, People’s Republic of China), with a high voltage applied to the blunt end; the distance from the sharp tip to the inlet of the MS was approximately 5 mm (***d***_***1***_). Extraction solvent (methanol, analytical reagent grade) was infused through a stainless steel capillary (internal diameter 0.18 mm) at a flow rate of 4 μL min^−1^ with a syringe pump (Harvard Apparatus, Model 11) and HPLC fittings. The distance between the capillary and the needle tip was 5 mm (***d***_***2***_; Fig. 1a). A ring stand and ring stand clamp hardware were used.

Ion source and the LTQ mass spectrometer were operated in positive ion detection modes. The electrospray ionization (ESI) voltage was +3.5 kV, and the temperature of the capillary inlet was 150 °C. The mass range was set at 50–2000 Da. Other parameters were set at instrument default values and no further optimization was performed. Five sampling signals were acquired from each tissue sample as the solvent dripped five times continuously. For collision-induced dissociation (CID), the MS/MS spectrum for ion detection used a maximum injection time of 100 ms. Ion Selection Operation (ISO) width was 1 Da, normalized collision energy was 13%, third grade collision energy was 14.5%, and the other parameters were set at default values of the instrument.

### Data analysis

Mass spectra were collected in single-stage MS, positive ion mode, and in the mass range of *m/z* 100–1000. Spectral data was binned using a 0.01 Th bin size for high-resolution experiments and a 1.0 Th bin size for low-resolution experiments, and both were stored in a structured query language (SQL) database (Oracle, Redwood City, CA, USA). The database contained both the full-known classification of every tissue specimen corresponding to the spectra, the World Health Organization (WHO) tumor type, and WHO grade^2^. PLS-LDA^32^ analysis of the mass spectral fingerprint data was performed using Matlab (version 7.11, Mathworks Inc, Natick, MA, USA). The mass spectra were treated as a matrix X, in which the rows and the columns corresponded to sample cases and *m/z* value variables, respectively. The mass spectral data expressed by relative abundance were directly used for the PLS-LDA^33^,^34^. The presence of human lung cancer tissue (cancer) and adjacent normal lung tissue (normal) data were analyzed by PLS-LDA with a goal to establish whether the separation between cancer and normal was significant by prediction of class. PLS-LDA models were constructed to establish the difference between the two groups; the PLS-LDA processing was performed as previously described^33^. When the PLS-LDA was completed, the misclassification rate and discrimination accuracy were exported to spreadsheets, and then Matlab was used to present the results of the statistical analysis for better visualization. Student’s *t* test was performed to prove the significance of those spectra with relative abundance above 20% in positive ion mode between cancerous and noncancerous tissue.

## Additional Information

**How to cite this article**: Wei, Y. *et al.* Tissue spray ionization mass spectrometry for rapid recognition of human lung squamous cell carcinoma. *Sci. Rep.* **5**, 10077; doi: 10.1038/srep10077 (2015).

## References

[b1] Siegel R. L., Miller K. D. & Jemal A. Cancer statistics, 2015. CA: Cancer. J. Clin. 65, 5–29 (2015). 25559415 10.3322/caac.21254

[b2] Detterbeck F. C., Boffa D. J. & Tanoue L. T. The new lung cancer staging system. CHEST. Journal . 136, 260–271 (2009). 10.1378/chest.08-0978 19584208

[b3] Mazzone P. Preoperative evaluation of the lung resection candidate. Cleve. Clin. J. Med. 79 Electronic Suppl 1, eS17–eS22 (2012). 22614960 10.3949/ccjm.79.s2.04

[b4] Leufkens A. M., van den Bosch M. A., van Leeuwen M. S. & Siersema P. D. Diagnostic accuracy of computed tomography for colon cancer staging: a systematic review. Scand. J. Gastroenterol. 46, 887–894 (2011). 21504379 10.3109/00365521.2011.574732

[b5] Winther C. & Graem N. Accuracy of frozen section diagnosis: a retrospective analysis of 4785 cases. Apmis. 119, 259–262 (2011). 21492225 10.1111/j.1600-0463.2011.02725.x

[b6] Nakhleh R. E. Quality in surgical pathology communication and reporting. Arch. Pathol. Lab. Med. 135, 1394–1397 (2011). 22032564 10.5858/arpa.2011-0192-RA

[b7] Xu X. *et al.* The accuracy of frozen section diagnosis of pulmonary nodules: evaluation of inflation method during intraoperative pathology consultation with cryosection. J. Thorac. Oncol. 5, 39–44 (2010). 19934776 10.1097/JTO.0b013e3181c09f9c

[b8] Takats Z., Denes J. & Kinross J. Identifying the margin: a new method to distinguish between cancerous and noncancerous tissue during surgery. Future. Oncol. 8, 113–116 (2012). 22335574 10.2217/fon.11.151

[b9] Liu J., Cooks R. G. & Ouyang Z. Biological tissue diagnostics using needle biopsy and spray ionization mass spectrometry. Anal. Chem. 83, 9221–9225 (2011). 22103750 10.1021/ac202626f PMC3245870

[b10] Zhang H. *et al.* Direct characterization of bulk samples by internal extractive electrospray ionization mass spectrometry. Sci. Rep. 3, 2495 (2013). 23970067 10.1038/srep02495 PMC3750536

[b11] Takats Z. Analysis of dried blood spot samples by high resolution mass spectrometry—from newborn screening to cancer diagnostics. Clin. Biochem. 47, 699 (2014). 24845710 10.1016/j.clinbiochem.2014.05.014

[b12] Lee do Y. *et al.* Resolving brain regions using nanostructure initiator mass spectrometry imaging of phospholipids. Integr. Biol. (Camb) . 4, 693–699 (2012). 22543711 10.1039/c2ib20043k PMC3698601

[b13] Smentkowski V. S. & Ostrowski S. G. Time of flight secondary ion mass spectrometry: a powerful high throughput screening tool. Rev. Sci. Instrum. 78, 072215 (2007). 17672746 10.1063/1.2755693

[b14] Mirnezami R. *et al.* Chemical mapping of the colorectal cancer microenvironment via MALDI imaging mass spectrometry (MALDI-MSI) reveals novel cancer-associated field effects. Mol. Oncol. 8, 39–49 (2014). 24112879 10.1016/j.molonc.2013.08.010 PMC5528498

[b15] Rujoi M., Estrada R. & Yappert M. C. *In situ* MALDI-TOF MS regional analysis of neutral phospholipids in lens tissue. Anal. Chem. 76, 1657–1663 (2004). 15018564 10.1021/ac0349680

[b16] Sachfer K. C. *et al.* *In situ*, real-time identification of biological tissues by ultraviolet and infrared laser desorption ionization mass spectrometry. Anal. Chem. 83, 1632–1640 (2011). 21302917 10.1021/ac102613m

[b17] Kerian K. S., Jarmusch A. K. & Cooks R. G. Touch spray mass spectrometry for *in situ* analysis of complex samples. Analyst. 139, 7 (2014). 10.1039/c4an00548a PMC4063212 24756256

[b18] Eberlin L. S. *et al.* Ambient mass spectrometry for the intraoperative molecular diagnosis of human brain tumors. Proc. Natl. Acad. Sci. USA 110, 1611–1616 (2013). 23300285 10.1073/pnas.1215687110 PMC3562800

[b19] Yoshimura K. *et al.* Real-time analysis of living animals by electrospray ionization, Analytical Biochemistry , 417, 195–206 (2011). 21741944 10.1016/j.ab.2011.06.020

[b20] Otsuka Y. *et al.* Imaging Mass Spectrometry of a mouse brain by tapping-mode scanning probe electrospray ionization. Analyst , 139, 2336–2341 (2014). 24683596 10.1039/c3an02340k

[b21] Peng I. X., Ogorzalek Loo R. R., Margalith E., Little M. W. & Loo J. A. Electrospray-assisted laser desorption ionization mass spectrometry (ELDI-MS) with an infrared laser for characterizing peptides and proteins. Analyst. 135, 767–772 (2010). 20349541 10.1039/b923303b PMC3006438

[b22] Harris G. A., Galhena A. S. & Fernandez F. M. Ambient sampling/ionization mass spectrometry: applications and current trends. Anal. Chem. 83, 4508–4538 (2011). 21495690 10.1021/ac200918u

[b23] Santagata S. *et al.* Intraoperative mass spectrometry mapping of an onco-metabolite to guide brain tumor surgery. Proc. Natl. Acad. Sci. USA 111, 11121–11126 (2014). 24982150 10.1073/pnas.1404724111 PMC4121790

[b24] Yang Q. *et al.* Paper spray ionization devices for direct, biomedical analysis using mass spectrometry. Int. J. Mass. Spectrom. 312, 201–207 (2012). 22350566 10.1016/j.ijms.2011.05.013 PMC3281765

[b25] Hu B., So P. K., Chen H. & Yao Z. P. Electrospray ionization using wooden tips. Anal. Chem. 83, 8201–8207 (2011). 21923155 10.1021/ac2017713

[b26] Li H., Liang Y., Xu Q. & Cao D. Key wavelengths screening using competitive adaptive reweighted sampling method for multivariate calibration. Anal. Chim. Acta. 648, 77–84 (2009). 19616692 10.1016/j.aca.2009.06.046

[b27] Jablonka S., Ledwozyw A., Kadziolka W. & Modrzewski Z. Phospholipids and fatty acids in pulmonary surfactant of patients with lung cancer. Pneumonol. Pol. 57, 270–276 (1989). 2633144

[b28] Snyder J. C., Teisanu R. M. & Stripp B. R. Endogenous lung stem cells and contribution to disease. J. Pathol. 217, 254–264 (2009). 19039828 10.1002/path.2473 PMC2773672

[b29] Ishii T. *et al.* Association between genetic variations in surfactant protein d and emphysema, interstitial pneumonia, and lung cancer in a japanese population. COPD . 9, 409–416 (2012). 22509983 10.3109/15412555.2012.676110

[b30] Sin D. D. *et al.* Pro-Surfactant protein B as a biomarker for lung cancer prediction. J. Clin. Oncol. 31, 4536–4543(2013). 24248694 10.1200/JCO.2013.50.6105 PMC3871515

[b31] Eberlin L. S. *et al.* Discrimination of human astrocytoma subtypes by lipid analysis using desorption electrospray ionization imaging mass spectrometry. Angew. Chem. Int. Ed. Engl. 49, 5953–5956 (2010). 20602384 10.1002/anie.201001452 PMC3021787

[b32] Wold S., Sjostrom M. & Eriksson L. PLS-regression: a basic tool of chemometrics. Chemometr. Intell. Lab. 58, 109–130 (2001).

[b33] Li H.-D., Liang Y.-Z., Xu Q.-S. & Cao D.-S. Model population analysis for variable selection. J. Chemometr. 24, 418–423 (2010).

[b34] Yi L. Z., He J., Liang Y. Z., Yuan D. L. & Chau F. T. Plasma fatty acid metabolic profiling and biomarkers of type 2 diabetes mellitus based on GC/MS and PLS-LDA. Febs. Lett. 580, 6837–6845 (2006). 17141227 10.1016/j.febslet.2006.11.043

